# Experimental Investigation of Principal Residual Stress and Fatigue Performance for Turned Nickel-Based Superalloy Inconel 718

**DOI:** 10.3390/ma11060879

**Published:** 2018-05-24

**Authors:** Yang Hua, Zhanqiang Liu

**Affiliations:** 1 Key Laboratory of High Efficiency and Clean Mechanical Manufacture of MOE, School of Mechanical Engineering, Shandong University, Jinan 250061, China; sduhuayang@gmail.com; 2 Key National Demonstration Center for Experimental Mechanical Engineering Education, Shandong University, Jinan 250061, China

**Keywords:** principal residual stress, surface residual stress, fatigue performance, Inconel 718, turning

## Abstract

Residual stresses of turned Inconel 718 surface along its axial and circumferential directions affect the fatigue performance of machined components. However, it has not been clear that the axial and circumferential directions are the principle residual stress direction. The direction of the maximum principal residual stress is crucial for the machined component service life. The present work aims to focuses on determining the direction and magnitude of principal residual stress and investigating its influence on fatigue performance of turned Inconel 718. The turning experimental results show that the principal residual stress magnitude is much higher than surface residual stress. In addition, both the principal residual stress and surface residual stress increase significantly as the feed rate increases. The fatigue test results show that the direction of the maximum principal residual stress increased by 7.4%, while the fatigue life decreased by 39.4%. The maximum principal residual stress magnitude diminished by 17.9%, whereas the fatigue life increased by 83.6%. The maximum principal residual stress has a preponderant influence on fatigue performance as compared to the surface residual stress. The maximum principal residual stress can be considered as a prime indicator for evaluation of the residual stress influence on fatigue performance of turned Inconel 718.

## 1. Introduction

Nickel-based superalloy Inconel 718 (IN718) has excellent mechanical properties and corrosion resistance even at high temperatures [1]. Thus, it has been extensively used in the aerospace industry for the hot-sections of gas turbine engines such as turbine disks [2,3]. Aero-engine turbine disks work in severe environments with high load, high temperature and high speed. Once the turbine disk has a fatigue fracture failure, the high-energy debris will be generated. These debris are unlikely to be contained by the turbine casting, which can threaten the aircraft safety significantly and may cause catastrophic accident. The turbine disk is thus classified as one of the fracture-critical parts of gas turbine engines.

Statistical results point out that the surface integrity is the largest cause of disk failures [4]. Among the surface integrity of a machined component, the relevant factors include residual stress, surface roughness, microhardness and microstructure. In particular, the residual stress plays a key role in the service life of machined components [5,6,7,8]. The compressive residual stress is in general effective in improving fatigue performance [9,10,11,12], whereas tensile residual stress is usually detrimental to fatigue life of machined components [13,14]. Consequently, residual stress should be taken into account at a high safety level against the all possible fatigue failures.

A number of researchers have worked on experimental investigations of the residual stress on machined surfaces. Pawade et al. [15] studied the residual stress along the circumferential direction of a machined surface with high-speed turning Inconel 718; their results showed that the tensile residual stresses increased along with the cutting speed increasing from 125 to 300 m/min, whereas the residual stresses changed from tensile to compressive when the cutting speed increased from 300 to 475 m/min. As the feed rate changed from 0.05 to 0.1 mm/rev there was a reversal of residual stresses from compressive to tensile, but a further increase in the feed rate to 0.15 mm/rev induced a small increase of tensile values. However, the residual stress along the axial direction was not taken into account in their experiments.

Arrazola et al. [16] analyzed the residual stress along axial and circumferential directions through turning Inconel 718. They found that the surface residual stresses tended to be higher along the circumferential direction than along the axial direction. The maximum compressive residual stress was higher along the circumferential direction. On the other hand, the results showed that both surface residual stresses and the maximum compressive residual stress along axial and circumferential directions were higher when the cutting speeds increased.

Madariaga et al. [17] focused on investigating the surface residual stress by means of face turning Inconel 718. Their research results revealed that the tensile residual stresses were generated on the machined surface along both the axial and circumferential directions. The surface residual stress was similar along the circumferential direction for all the investigated cases. The surface residual stress increased along the axial direction when increasing the cutting speed or the feed rate. 

Berruti et al. [18] focused on establishing a reliable experimental database of residual stress by turning Inconel 718. They analyzed the residual stresses along the axial and circumferential directions. They found that the surface residual stress along the axial direction was more sensitive to the cutting parameters than that along the circumferential direction. In addition, the results indicated that the surface residual stresses tended to be always tensile along both these two directions.

In summary, most previous studies focused on the effect of cutting parameters on residual stresses along the two directions of circumferential and axial direction. However, there have been few pieces of research that have demonstrated that these two directions are the principal stress direction [19,20]. It has been well acknowledged that the principal stresses represent the stress state of one point in a solid body. When the principal stress is determined, it is possible to determine the direction in which the maximum stress is located. The magnitude and direction of the principal stress determine the failure of machined components. If the direction of the maximum principal stress coincides with the direction of external load that applies to the machined component, the maximum principal stress will add up to the external load during the service of machined component. This will lead to the earlier failure of the machined component. Conversely, if the direction of maximum principal stress is opposite to the external load, the failure of the component will be delayed. Thus, the research of direction and magnitude of the principal residual stress has a huge importance, especially in the aerospace industry. 

The purpose of this work is to determine the direction and magnitude of principal residual stress of machined components. The effect of principal residual stress on fatigue performance of Inconel 718 specimen is investigated. Firstly, turning experiments of Inconel 718 are analyzed based on surface residual stress and the maximum principal residual stress. Then, the fatigue performance of turned specimens by fatigue tests are discussed. The mechanism of surface residual stress and the maximum principal residual stress on fatigue behavior for turned specimens are revealed.

## 2. Experiments

### 2.1. Materials and Turning Experiments

Through solution heat treatment (heat treating at 960 °C for 1 h, followed by air-cooling) and aging treatment (heat treating at 720 °C for 8 h, then furnace cooling to 620 °C with a cooling rate of 50 °C/h, aging for 8 h, and finally air-cooling to room temperature) Inconel 718 was employed as the workpiece material in this paper. The chemical compositions, mechanical properties and specific heat capacity of this alloy are shown in Table 1, Table 2 and Table 3, respectively.

A set of cylindrical turning experiments were carried out to induce different residual stresses by varying cutting speeds and feed rates. The turning process was performed without cutting fluid, and the cutting conditions employed in the experiments are summarized in Table 4. All turning tests were conducted on a CNC turning center with the maximum spindle speed of 6000 rpm and a power rating of 28.66 KW. The carbide inserts ISO VBMT110308-1105 with PVD coating (TiAlN) and a tool holder ISO SVJBR 2525M11 were employed for turning experiments. Each turning experiment was conducted using a fresh sharp tool to eliminate the influence of worn tools on residual stress in the present work.

### 2.2. Fatigue Tests

**Figure 1:**
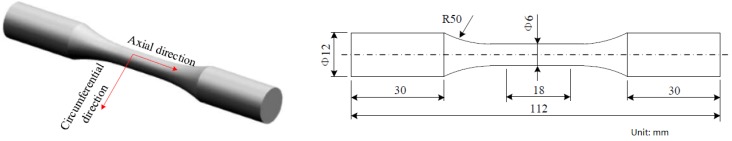
Shape and dimensions of fatigue test specimen.

Fatigue tests were performed to investigate the effect of principal residual stress on fatigue performance of machined components. Although Hua et al. [21] suggested that the cutting speed was not the dominant factor influencing the surface roughness and Moussaoui et al. [22,23] demonstrated that the surface roughness *R_a_* had no influence on the fatigue life, the effect of surface roughness on fatigue performance was eliminated by two steps in this work. Firstly, the specimens machined under the lower feed rate 0.075 mm/rev were chose in fatigue tests, because the feed rate was the dominant factor affecting the surface roughness [24,25]. Then the machined specimens with lower feed rate used in fatigue tests were polished by hand to remove the scratches and the concentration of stress generated during machining. The axial fatigue tests were conducted on a high-frequency fatigue testing machine PLG-100 (Letry, Xi’an, China) at room temperature. The dimensions of fatigue test specimens were shown in Figure 1. During the fatigue tests, a dynamic sinusoidal load was applied on the specimens, and the maximum tension stress was 1237 MPa. The loading frequency was 108~114 Hz, and the cycle stress ratio *R* was set 0.1. When the specimen fractured in the fatigue test, the number of cycles was recorded as its fatigue life. At least three specimens were tested for each cutting condition to eliminate the random error in the present work.

## 3. Residual Stress Measurements

**Figure 2:**
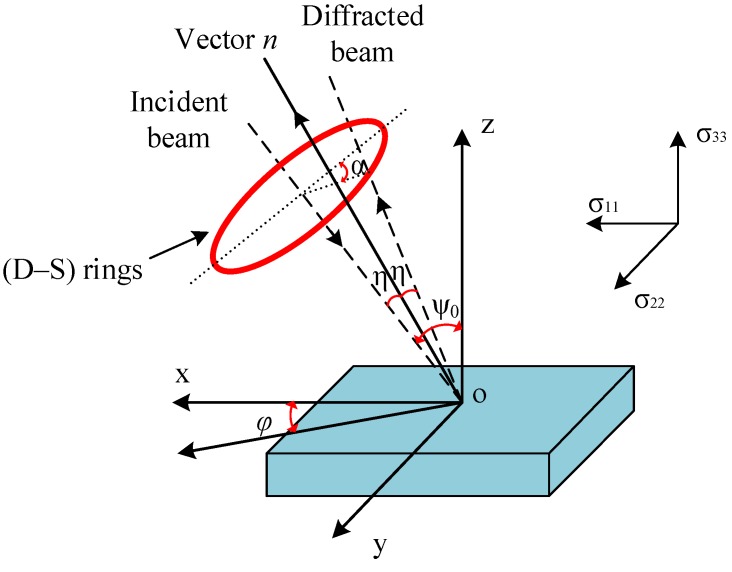
Schematic diagram of Debye-Scherrer ring measurement.

The measurements of residual stress were carried out with cos*α* method [26] by means of X-ray diffraction technique. A Pulstec μ-X360n (Pulstec, Hamamatsu, Japan) residual stress analyzer with a Cr-K β radiation (Pulstec, Hamamatsu, Japan) was utilized for the stress determination. Residual stresses were measured in three different directions on the machined surface (see Figure 2): circumferential direction (cutting direction, *φ* = 90°), axial direction (feed direction, *φ* = 0°) and an intermediate direction (*φ* = 45°), respectively. The strain, *ε_α_*, for a crystallographic plane (311) can be determined from the change of the diffraction angle given by the radius of Debye-Scherrer ring (D-S ring) [26] at an angle *α* shown in Figure 2. The measurements of strain at four directions (*α*, *π* + *α*, −*α*, *π* − *α*) on the D-S ring to calculate the average strain $\bar{\varepsilon_{\alpha }}$. *ε_α_* and $\bar{\varepsilon_{\alpha }}$ are expressed by Equations (1) and (2).

(1) $$\varepsilon_{\alpha }=\frac{d_{1}-d_{0}}{d_{0}}=n_{1}^{2}\varepsilon_{xx}+n_{2}^{2}\varepsilon_{yy}+n_{3}^{2}\varepsilon_{zz}+n_{1}n_{2}\gamma_{xy}+n_{2}n_{3}\gamma_{yz}+n_{1}n_{3}\gamma_{xz}$$

(2) $$\bar{\varepsilon_{\alpha }}=\left[\left(\varepsilon_{\alpha }-\varepsilon_{\pi +\alpha }\right)+\left(\varepsilon_{-\alpha }-\varepsilon_{\pi -\alpha }\right)\right]/2$$

where, *d*_0_ and *d*_1_ are the interplanar spacing for an unstressed specimen and the interplanar spacing obtained from the position of the diffraction peak, respectively. *d*_0_, *d*_1_ can be obtained with Bragg law by Equation (3).

(3) λ=2d⋅sinθ

where, *λ* is the wavelength and *θ* is the Bragg angle.

The parameters *n*_1_, *n*_2_, *n*_3_ in Equation (1) are the direction cosines of diffraction vector **n**. *n*_1_, *n*_2_, *n*_3_ can be expressed as Equation (4)

(4) $$n=\left(\begin{array}{c} n_{1}\\ n_{2}\\ n_{3} \end{array}\right)=\left(\begin{array}{c} \cos\eta \sin\psi_{0}\cos\phi -\sin\eta \cos\psi_{0}\cos\phi \cos\alpha -\sin\eta \sin\phi \sin\alpha \\ \cos\eta \sin\psi_{0}\sin\phi -\sin\eta \cos\psi_{0}\sin\phi \cos\alpha +\sin\eta \cos\phi \sin\alpha \\ \cos\eta \cos\psi_{0}+\sin\eta \sin\psi_{0}\cos\alpha \end{array}\right)$$

where, 2*η* is the semi-angle of D-S ring, *φ* and *ψ* angles are defined as the in-plane direction, and the angle between specimen normal and incident beam, respectively, as shown in Figure 2.

As the penetration depth of X-rays in nickel-based superalloy Inconel 718 is approximately 5 μm, the plane stress model can thus be assumed. Based on the theory of elastic mechanic, *ε_xx_, ε_yy_, ε_zz_, γ_xy_, γ_yz_, γ_zx_* can be expressed with Equation (5).

(5) $$\varepsilon_{xx}=\frac{1}{E}\left(\sigma_{xx}-\nu \sigma_{yy}\right)\;\varepsilon_{yy}=\frac{1}{E}\left(\sigma_{yy}-\nu \sigma_{xx}\right)\;\varepsilon_{\mathrm{zz}}=-\frac{\nu }{E}\left(\sigma_{xx}+\nu \sigma_{yy}\right)\;\gamma_{xy}=\frac{2\left(1+\nu \right)}{E}\tau_{xy}\;\gamma_{yz}=\gamma_{zx}=0$$

where, *ν* and *E* are the Poisson’s ratio and the Young’s modulus of the material, respectively. *σ_xx_, σ_yy_* and *σ_xy_* are the stress tensor component of a point in the solid body. Accordingly, Equation (2) can be written as Equation (6).

(6) $$\bar{\varepsilon_{\alpha }}=-\frac{1+\nu }{E}\sigma_{\phi }\sin2\psi_{0}\sin2\eta \cos\alpha$$

where, *σ_φ_* is the in-plane stress. The stress can be calculated by varying *α* from 0° to 90° to cover the whole ring and is a linear function of the regression between $\bar{\varepsilon_{\alpha }}$ and cosα. As a consequence, *σ_φ_* can be expressed by Equation (7).

(7) $$\sigma_{\phi }=-\frac{E}{1+\nu }\frac{1}{\sin2\eta }\frac{1}{\sin2\psi_{0}}\left(\frac{\partial \bar{\varepsilon_{\alpha }}}{\partial \cos\alpha }\right)$$

Based on the theory of X-ray and the elastic mechanics, the relationship between stress tensor component and *σ_φ_* can be expressed with Equation (8) [27,28] in plane stress condition.

(8) $$\sigma_{\phi }=\sigma_{11}\cos^{2}\phi +\sigma_{12}\sin2\phi +\sigma_{22}\sin^{2}\phi$$

where, *φ* is defined as in-plane direction as shown in Figure 1, *σ*_11_ = *σ_xx_*, *σ*_22_ = *σ_yy_* and *σ*_12_ = *σ_xy_*. Therefore, the stress tensor components (*σ*_11_, *σ*_22_, *σ*_12_) can be obtained through the measured residual stresses along the three different directions *φ*. 

**Figure 3:**
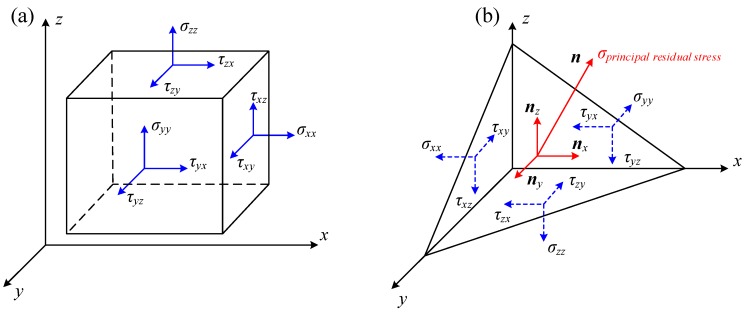
Residual stress for one point in solid body (**a**) normal residual stresses and (**b**) principal residual stress.

As analyzed above, the residual stresses measured by means of the X-ray diffraction are normal residual stresses instead of principal residual stresses. As mentioned in introduction, the stress state of one point in solid body can be depicted by the principal residual stress. Once the normal residual stresses are obtained as shown in Figure 3a, the principal residual stress which indicates the direction of maximum stress of one point in solid body can be determined (see Figure 3b).

Based on the theory of elastic mechanics, the stresses on principal plane can be expressed by Equations (9) and (10).

(9) $$\sigma =\frac{\sigma_{xx}+\sigma_{yy}}{2}+\frac{\sigma_{xx}-\sigma_{yy}}{2}\cdot \cos2\alpha_{0}-\sigma_{xy}\cdot \sin2\alpha_{0}$$

(10) $$\tau =\frac{\sigma_{xx}-\sigma_{yy}}{2}\cdot \sin2\alpha_{0}+\sigma_{xy}\cdot \cos2\alpha_{0}$$

where, *σ* and *τ* are the normal stress and shear stress on the principal plane, respectively. *α*_0_ is the angle between the normal line of principal plane and *x*-axis. According to the definition of principal stress, the magnitude *σ*_principal_ and the direction *α*_principal_ of principal residual stress can be obtained by Equations (11) and (12).

(11) $$\sigma_{principal}=\frac{\sigma_{xx}+\sigma_{yy}}{2}\pm \sqrt{{\left(\frac{\sigma_{xx}-\sigma_{yy}}{2}\right)}^{2}+\sigma_{xy}^{2}}$$

(12) $$\alpha =\frac{1}{2}\arctan\frac{-2\sigma_{xy}}{\sigma_{xx}-\sigma_{yy}}$$

## 4. Results and Discussion

### 4.1. Surface Residual Stress

**Figure 4:**
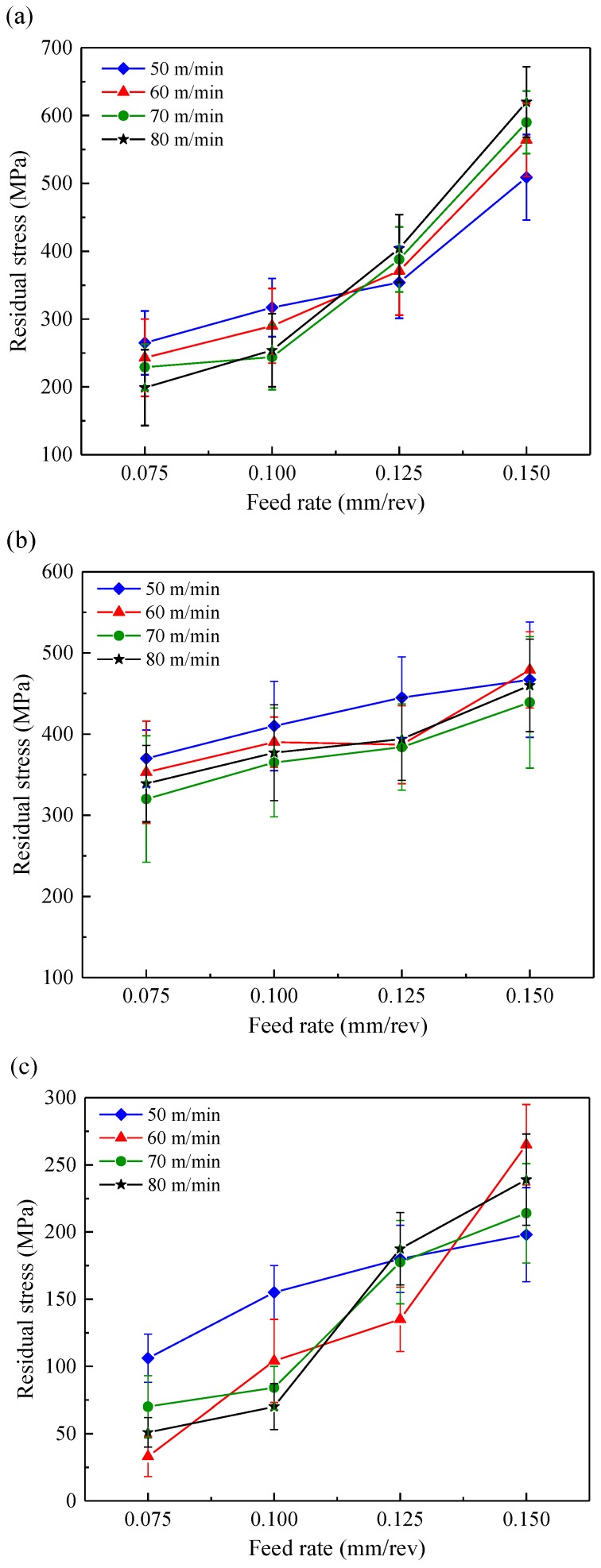
Surface residual stress along (**a**) axial direction, (**b**) circumferential direction and (**c**) intermediate direction.

The surface residual stresses measured along three directions—axial direction, circumferential direction and intermediate direction—in each test are summarized in Figure 4. It can be seen from Figure 4c that the residual stresses measured along intermediate directions were lower than those measured along the axial direction and circumferential direction. The surface residual stresses along the axial and circumferential directions for the whole cases in this research were tensile ones with values ranging from 199 MPa to 620 MPa depending upon the cutting condition employed. The highest values of surface residual stress along axial direction and circumferential direction were 620 MPa and 479 MPa, respectively.

As shown in Figure 4, the surface residual stress along the three directions (axial direction, circumferential direction and intermediate direction) increased significantly with the feed rate changing from 0.075 mm/rev to 0.15 mm/rev. This was in agreement with Sharman et al. [29] who found that a higher feed rate resulted in increased tensile residual stress of the machined surface when turning Inconel 718 with various feed rates. Madariaga et al. [14] suggested that the surface residual stress was similar in the cutting direction for all the analyzed cases but it increased in the feed direction when increasing the cutting speed or feed rate. However, in the present work the cutting speed appeared to have no influence on surface residual stress along circumferential direction as shown in Figure 4b. The tendency towards more tensile residual stress can be related to an increase of plastic deformation as the feed rate increased.

**Figure 5:**
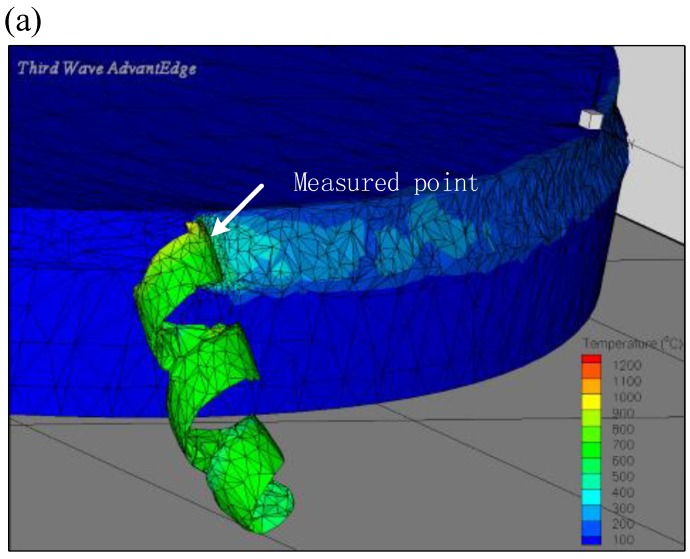

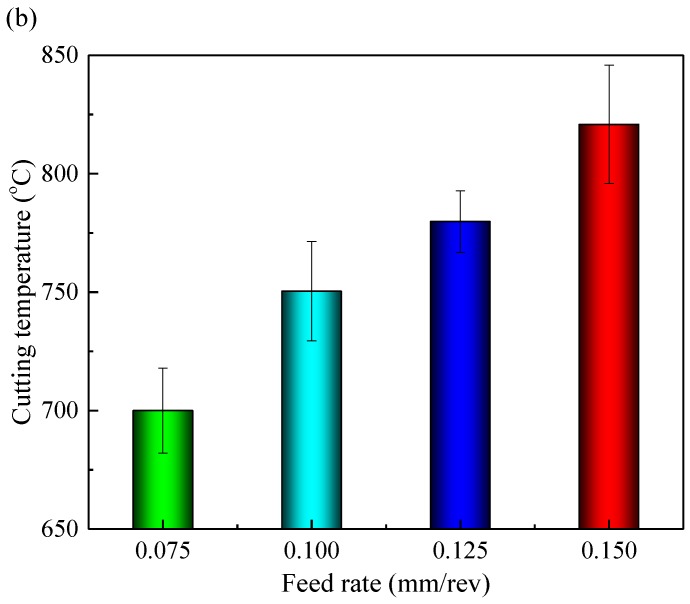
Three-dimensional finite element model (**a**) and cutting temperatures at different feed rates for the fixed cutting speed of 80 m/min (**b**).

**Figure 6:**
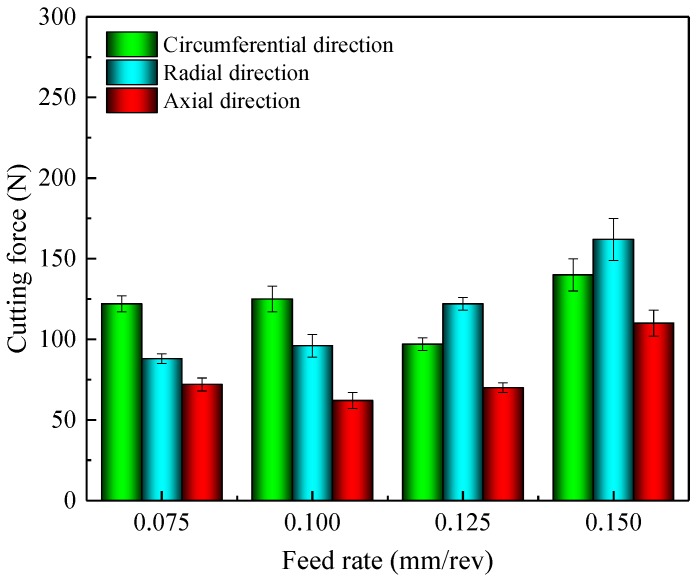
Cutting forces at different feed rates for the fixed cutting speed of 80 m/min.

**Figure 7:**
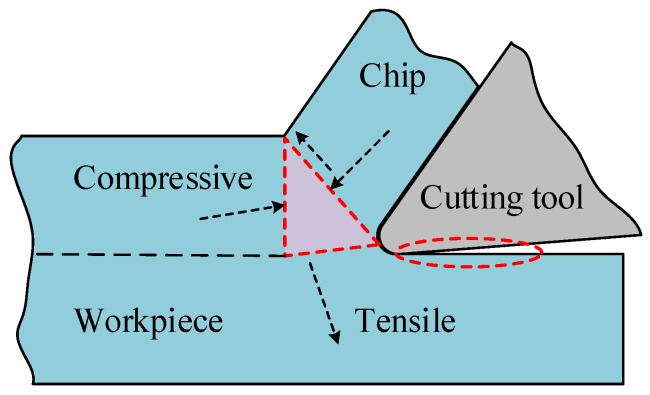
Schematic diagram of non-uniform plastic deformation generation during machining.

The cutting temperature in cutting zone was obtained from the finite element method (FEM) simulation model which was performed with a commercial software AdvantEdge. The three-dimensional finite element models were established to simulate the cutting process. The hardness of workpiece materials, the type of cutting tools and the cutting conditions were should be coincided with those at used in the experiments. The cutting temperature generated on the workpiece surface in the front of cutting zone was measured (see Figure 5a) and further summarized in Figure 5b. It can be seen that the cutting temperature increased rapidly when the feed rate increased. The cutting force measured during turning remained stable (see Figure 6). The residual stress mainly resulted from the non-uniform plastic deformation in the machined surface layer, and this non-uniform deformation was determined by the mechanical and thermal loads. During machining, the workpiece material ahead of the cutting tool induced compressive plastic deformation due to the compressive force, and the tensile plastic deformation was generated due to the shear force (as illustrated in Figure 7). In addition, the tensile plastic deformation was generated due to the squeezing and rubbing from the tool flank face. The tensile residual stress could be generated on the machined surface and vice versa when the total compressive plastic deformation was greater than the level of tensile plastic deformation. Meanwhile, the heat was generated due to chip formation in the primary shear zone and the friction between the workpiece surface and tool flank face during the cutting process. The cutting temperature was elevated with the increase of feed rate (see Figure 5), which resulted due to the higher specific heat capacity (see Table 3) of Inconel 718. Owing to the low thermal conductivity (13.4 W/(m·°C)) of Inconel 718, a large percentage of heat was transmitted into the machined surface. The amount of heat induced compressive plastic deformation on the machined workpiece surface due to the localized thermal expansion, which led to the surface tensile residual stress generation after rapid cooling. The final residual stress state could be determined by the interaction of all these factors and the thermal properties of the workpiece material. The results of this work show that the surface residual stress under all the cutting conditions used in this paper were tensile residual stress. It can be deduced that the total compressive plastic deformation was greater than the total tensile plastic deformation during machining. The larger compressive plastic deformation resulted in more tensile residual stress when the feed rate increased from 0.075 mm/rev to 0.15 mm/rev.

### 4.2. Principal Residual Stress

**Figure 8:**
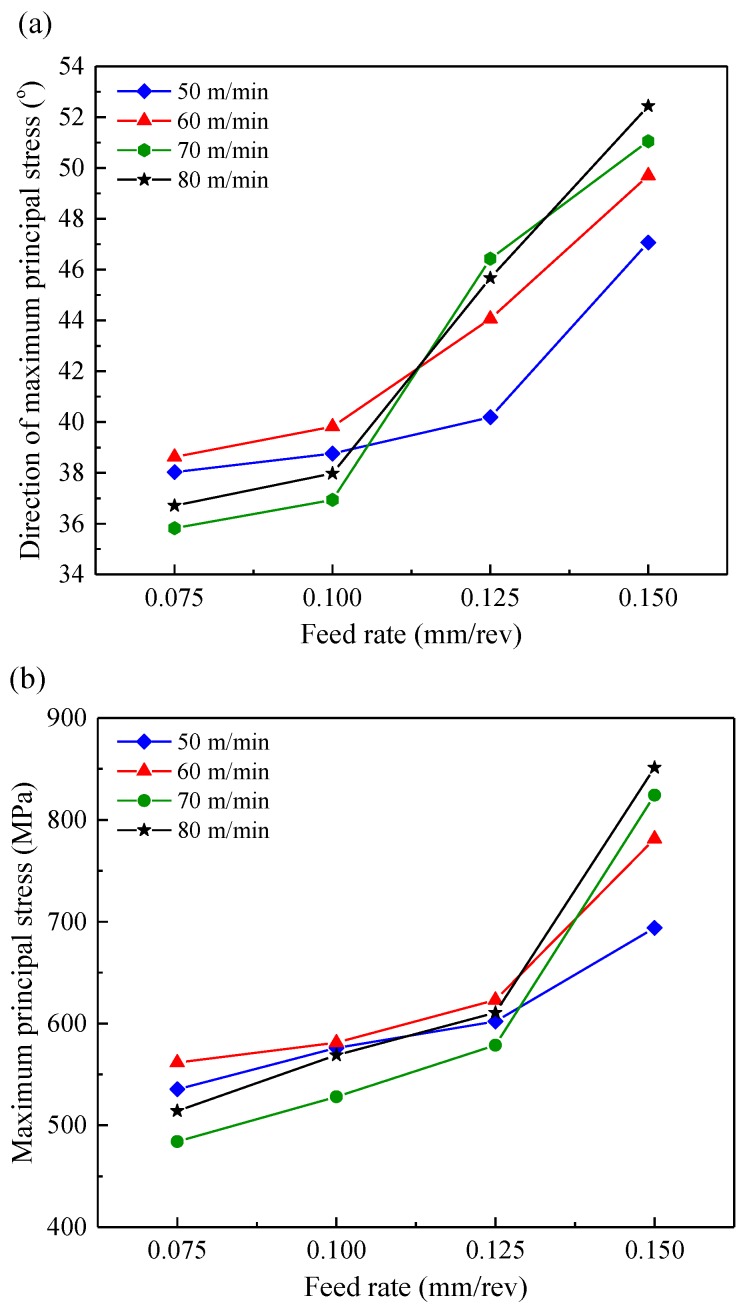
The maximum principal residual stress (**a**) direction and (**b**) magnitude.

The direction and magnitude of the maximum principal residual stress under all the cutting conditions in this research are summarized in Figure 8. Figure 8a shown that the maximum principal residual stress was located within the range of 36.4°~52.4° from the circumferential direction (the direction of the minimum principal residual stress was perpendicular to the maximum principal residual stress). The angle between the maximum principal residual stress and the circumferential direction was defined as the direction of maximum principal residual stress. It can be observed from Figure 8a that the maximum principal residual stress angle increased rapidly as the feed rate increased. It indicated that the direction of maximum principal stress tended to approach the axial direction with the increase of feed rates. This could be explained by the more increased surface residual stress along the axial direction than that along the circumferential direction. As shown in Figure 8b, the magnitude of the maximum principal residual stress increased significantly with the feed rate changing from 0.075 mm/rev to 0.15 mm/rev. This can be attributed to the increased surface residual stress along the axial direction and circumferential direction as explained in the previous section of this paper.

### 4.3. Effect of Principal Residual Stress on Fatigue Performance

**Figure 9:**
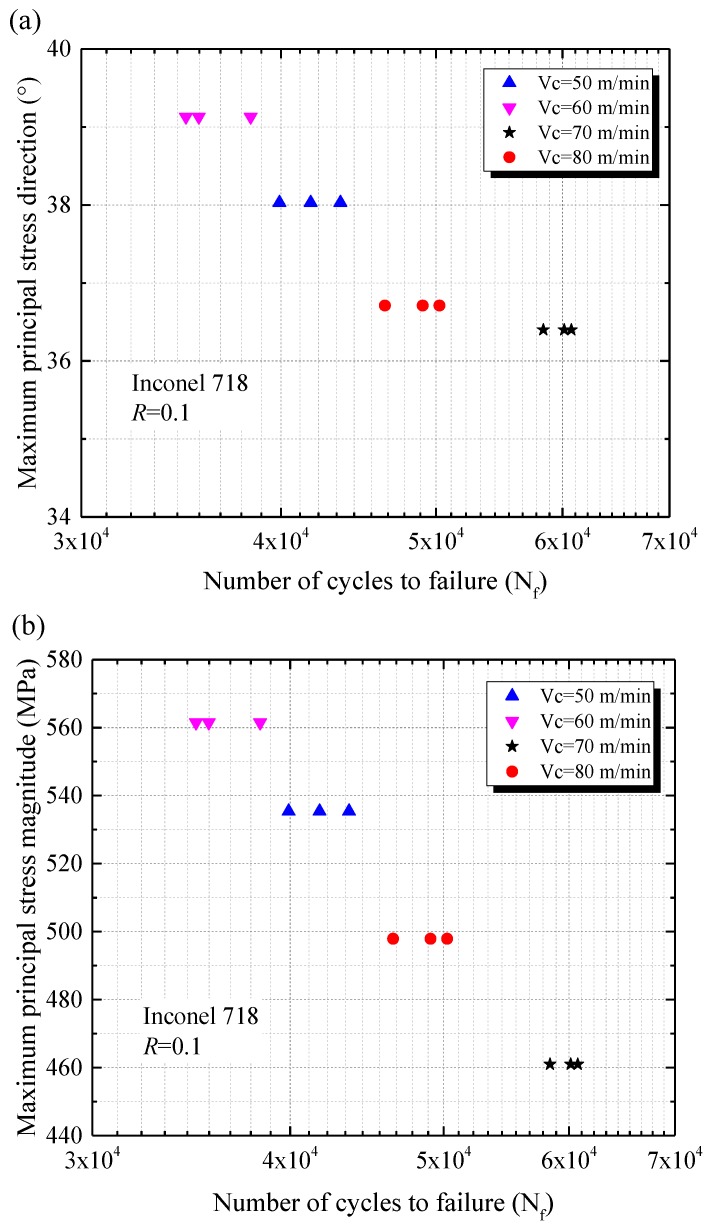
Sample fatigue life at different levels of the maximum principal residual stress (**a**) direction and (**b**) magnitude.

**Figure 10:**
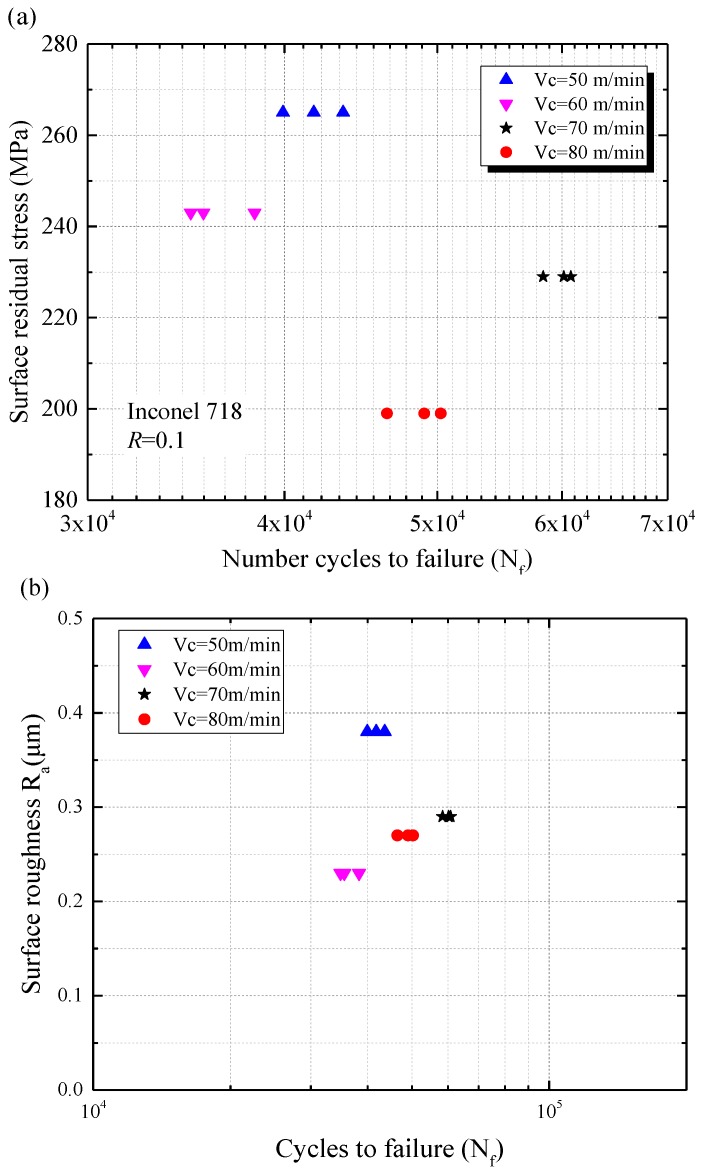
Sample fatigue life at different (**a**) surface residual stresses and (**b**) surface roughness.

Axial tension-tension fatigue tests were carried out to investigate the relationship between the maximum principal residual stress and the fatigue performance of machined specimens. Depending on the same loading condition, the fatigue performances at different levels of the maximum principal residual stress were shown in Figure 9. Fatigue test results show that the total fatigue life of the specimen was strongly dependent on the direction of the maximum principal residual stress. The highest and the lowest fatigue life occurred at the direction of the maximum principal residual stress were 36.4° and 39.1°, respectively (see Figure 9a). As the direction of the maximum principal residual stress increased from 36.4° to 39.1° (increased by 7.4%), the fatigue life decreased from 59,765 cycles to 36,236 cycles (decreased by 39.4%). It can be observed from Figure 9b that the magnitude of maximum principal residual stress affected significantly the fatigue life of the specimens. The highest and the lowest magnitude of the maximum principal residual stress were 561.5 MPa and 461.0 MPa, which corresponded to the lowest and the highest fatigue life of the specimens were 59,765 cycles and 36,236 cycles, respectively. It was noted that the maximum principal residual stress magnitude diminished by 17.9%, and the fatigue life increased by 83.6%. Figure 10a illustrates the relationship between fatigue life and surface residual stress along the axial direction. Figure 10b shows the relationship between fatigue life and surface roughness. However, there was no significant influence of surface roughness *R_a_* on the fatigue life under the conditions used in our research (see Figure 10b). This was consistent with the research of Moussaoui et al. [22,23]. The results shown that the fatigue life had almost no change with the increase of surface roughness and the surface residual stress along the axial direction. The fatigue tests demonstrated that the surface residual stress did not influence the fatigue life directly. It can be considered that the surface residual stress was not a suitable indicator to show the influence of residual stress on fatigue performance. The maximum principal residual stress may be the dominant factor affecting fatigue life of machined components.

The tendency of fatigue life to be much lower can be attributed to an increase in total stress at the axial direction. As mentioned in the introduction of this paper, it is of huge significance to know the direction and magnitude of the maximum principal residual stress. If the applied stress acts on the component during service in the same direction as the maximum principal residual stress, the total stress on the machined component surface can be defined by Equation (13).

(13) $$\sigma_{total\;stress}=\sigma_{applied\;stress}+\sigma_{\max\;principal\;stress}$$

If the direction of applied stress is not identical with the maximum principal residual stress, the total stress on the machined surface can be expressed with Equation (14).

(14) $$\sigma_{total\;stress}=\sigma_{applied\;stress}+\sigma_{\max\;principal\;stress}\cdot \cos\beta$$

where *β* is the angle between the maximum principal residual stress and the applied stress. In case of the total stress is greater than the yield stress of material, the plastic deformation is generated, which will result in the fatigue failure under the cycle loading. In the present work, the applied stress acts on the specimen is the cycle loading along the axial direction. Thus, the total stress can be obtained by Equation (15)

(15) $$\sigma_{total\;stress}=\sigma_{applied\;stress}+\sigma_{\max\;principal\;stress}\cdot \sin\alpha$$

where *α* is the direction of maximum principal residual stress (the angle between maximum principal residual stress and circumferential direction). Accordingly, the total stress acts on the machined component becomes higher with the increase of magnitude and direction of the maximum principal residual stress.

The maximum principal residual stress was generated by machining mainly has an effect on the fatigue life. This phenomenon can be explained by improving the effect of mean stress shown in Equation (16) [30].

(16) $$\frac{\Delta \sigma }{2}=\left({\sigma^{\prime }}_{f}-\sigma_{m}\right)\cdot {\left(2N_{f}\right)}^{b}$$

where Δ*σ* is the stress range, *σ′_f_* is the fatigue strength coefficient. *σ_m_* is the mean stress and *b* is the fatigue strength exponent. The maximum principal residual stress was tensile stress; however, the higher mean stress *σ_m_* was induced by the increased maximum principal residual stress. This could result in the fatigue life declining, according to Equation (16). Conversely, if the maximum principal residual stress is a compressive one, the raised maximum principal residual stress would lower the effect of the mean stress. The fatigue life thus can be prolonged during service. The direction and magnitude of maximum principal residual stress play a key role on the fatigue life. The maximum principal residual stress on the surface can be more suitable for indicating the effect of residual stress on fatigue performance of machined components.

## 5. Conclusions

In the present work, both the direction and the magnitude of principal residual stresses were determined with cos*α* method. The axial tension-tension fatigue tests were performed on the machined Inconel 718 specimens. The surface residual stresses along the axial and circumferential directions, the direction and magnitude of principal residual stresses were presented and analyzed. The influence of the maximum principal residual stress on fatigue performance of Inconel 718 specimens was revealed. From the results obtained in this work and based on the current knowledge of mechanics and fatigue of materials, the following conclusions can be derived:

- As the feed rate increases, the surface residual stress tends to be more tensile. The tendency towards more tensile residual stress is related to the larger compressive deformation induced by higher feed rates.
- The maximum principal residual stress direction and magnitude increased significantly with the increase of feed rate. The magnitude of the maximum principal residual stresses was much higher than those of surface residual stresses along the axial and circumferential direction.
- The direction and magnitude of the maximum principal residual stress tended to be higher, which implied that the direction of the maximum principal residual stress approached the axial direction. The larger total stress in the axial direction was generated, which resulted in lower fatigue life.
- As the direction of the maximum principal residual stress increased from 36.4° to 39.1° (increased by 7.4%), the fatigue life decreased from 59,765 cycles to 36,236 cycles (decreased by 39.4%). The maximum principal residual stress magnitude diminished by 17.9%, and the fatigue life increased by 83.6%.
- The maximum principal residual stress had a dominant influence on fatigue life compared to that of the surface residual stress along the axial direction. Fatigue tests demonstrated that the surface residual stress appeared to have no influence on fatigue life. The maximum principal residual stress can be considered a prime indicator for evaluation of the residual stress impact on the fatigue performance of machined components.

## Figures and Tables

**Table 1:** Chemical compositions of Inconel 718.

Element	Fe	Cr	Nb	Mo	Ti	Al	Co	Ni
wt %	18.19	18.05	5.43	2.98	1.02	0.50	0.31	Balance

**Table 2:** Mechanical properties of Inconel 718.

Work Temperature (°C)	Tensile Strength *σ*_b_ (MPa)	Yield Strength *σ*_0.2_ (MPa)	Elongation (%)	Shrinkage (%)	Hardness (HBW)
20	1502	1360.5	19.3	34.5	439

**Table 3:** Specific heat capacity of Inconel 718 at different temperatures.

Temperature (°C)	300	400	500	600	700	800
Specific heat capacity (J/(kg·°C))	481.4	493.9	514.8	539.0	573.4	615.3

**Table 4:** Turning conditions for turning Inconel 718.

Parameters	Cutting Speed (m/min)	Feed Rate (mm/rev)	Nose Radius (mm)	Depth of Cut (mm)
Levels	50, 60, 70, 80	0.075, 0.10, 0.125, 0.15	0.8	0.2
